# Smoking, tobacco dependence, and neurometabolites in the dorsal anterior cingulate cortex

**DOI:** 10.1038/s41380-023-02247-0

**Published:** 2023-09-25

**Authors:** Joseph O’Neill, Maylen Perez Diaz, Jeffry R. Alger, Jean-Baptiste Pochon, Dara Ghahremani, Andrew C. Dean, Rachel F. Tyndale, Nicole Petersen, Shane Marohnic, Andrea Karaiskaki, Edythe D. London

**Affiliations:** 1 Division of Child & Adolescent Psychiatry, Department of Psychiatry, University of California at Los Angeles, Los Angeles, CA USA; 2 Brain Research Institute, University of California at Los Angeles, Los Angeles, CA USA; 3 Jane and Terry Semel Institute for Neuroscience and Human Behavior and the Department of Psychiatry, University of California at Los Angeles, Los Angeles, CA USA; 4 Biogen, Inc., Nashville, TN USA; 5 Department of Neurology, University of California at Los Angeles, Los Angeles, CA USA; 6 Department of Pharmacology & Toxicology, and Department of Psychiatry, University of Toronto, and Campbell Family Mental Health Research Institute, Centre for Addiction & Mental Health, Toronto, ON Canada

**Keywords:** Addiction, Prognostic markers, Neuroscience

## Abstract

Cigarette smoking has a major impact on global health and morbidity, and positron emission tomographic research has provided evidence for reduced inflammation in the human brain associated with cigarette smoking. Given the consequences of inflammatory dysfunction for health, the question of whether cigarette smoking affects neuroinflammation warrants further investigation. The goal of this project therefore was to validate and extend evidence of hypoinflammation related to smoking, and to examine the potential contribution of inflammation to clinical features of smoking. Using magnetic resonance spectroscopy, we measured levels of neurometabolites that are putative neuroinflammatory markers. *N*-acetyl compounds (*N*-acetylaspartate + *N*-acetylaspartylglutamate), glutamate, creatine, choline-compounds (phosphocholine + glycerophosphocholine), and *myo*-inositol, have all been linked to neuroinflammation, but they have not been examined as such with respect to smoking. We tested whether people who smoke cigarettes have brain levels of these metabolites consistent with decreased neuroinflammation, and whether clinical features of smoking are associated with levels of these metabolites. The dorsal anterior cingulate cortex was chosen as the region-of-interest because of previous evidence linking it to smoking and related states. Fifty-four adults who smoked daily maintained overnight smoking abstinence before testing and were compared with 37 nonsmoking participants. Among the smoking participants, we tested for associations of metabolite levels with tobacco dependence, smoking history, craving, and withdrawal. Levels of *N*-acetyl compounds and glutamate were higher, whereas levels of creatine and choline compounds were lower in the smoking group as compared with the nonsmoking group. In the smoking group, glutamate and creatine levels correlated negatively with tobacco dependence, and creatine correlated negatively with lifetime smoking, but none of the metabolite levels correlated with craving or withdrawal. The findings indicate a link between smoking and a hypoinflammatory state in the brain, specifically in the dorsal anterior cingulate cortex. Smoking may thereby increase vulnerability to infection and brain injury.

## Introduction

Approximately 1.3 billion people smoke cigarettes worldwide, and there are about eight million annual smoking-related deaths [1], attributed to oncologic, respiratory, cardiovascular, and other causes [2]. The harms related to smoking likely include multiple aspects of inflammatory dysfunction. Smoking produces reactive oxygen species that promote oxidative stress and inflammation leading to carcinogenesis [3]. However, in the brain and other organs, inflammation protects against infection and aids recovery from injury [4], and there is evidence that people who smoke exhibit an attenuated inflammatory healing response [5, 6]. Positron emission tomographic (PET) studies recently added in vivo evidence of a hypoinflammatory state throughout the brains of people who smoke [7, 8]. Those studies measured the uptake of a radiotracer for the 18 kDa translocator protein (TSPO), a marker for inflammation that labels activated microglia [9]. Given the ubiquity of TSPO in the brain, especially at the blood-brain barrier [10] and its interaction with numerous ligands, such as cholesterol [11], additional studies that use alternative methods to assess potential smoking-related effects on neuroinflammation are warranted.

As detailed in several reviews [12–15], neuroinflammation may be detectable by metabolites measured with proton magnetic resonance spectroscopy (MRS). Levels of *N*-acetylaspartate + *N*-acetylaspartylglutamate (*N*-acetyl compounds, NAA) and glutamate (Glu) are diminished in wide-ranging inflammatory neurological conditions, whereas levels of creatine + phosphocreatine (Cr), choline-compounds (Cho), and *myo*-inositol (mI) are elevated. Thus, levels of these metabolites in the brain may inform questions regarding neuroinflammation in smoking and its clinical correlates.

We selected the dorsal anterior cingulate cortex (dACC) as a region-of-interest to examine this issue because functional magnetic resonance imaging (fMRI) findings have implicated the dACC in smoking-related behavioral states [16, 17]. Relevant evidence includes greater reactivity to smoking-related cues in the dACC of patients who lapsed after initiating smoking cessation compared to those who maintained abstinence [18], as well as lower activation in the dACC following behavioral therapy that diminished tobacco craving [19] and following presentation of smoking-aversive cues [20]. In addition, resting-state functional connectivity of the dACC with the insula was positively correlated with the severity of tobacco withdrawal [21].

Several prior MRS studies related to smoking have involved glutamatergic compounds in the dACC. Some of those findings were reported as Glu, others as Glx (the sum of Glu with glutamine, Gln), and still others as Glu or Glx normalized to creatine (Glu/Cr or Glx/Cr). The choice of glutamatergic endpoint has had no apparent impact on the results. Higher Glx and Glx/Cr were found in people who smoked than those who did not [22, 23], and Glx was higher during smoking satiety as compared with abstinence [22, 24]. However, one study found higher Glx and Glu and stronger functional connectivity to other cortices during tobacco withdrawal than during satiety [24]. These results are inconsistent, indicating the need for further rigorous MRS studies of the dACC, as we have attempted here. Finally, the aforementioned reduction in binding to TSPO (a marker for inflammation) among smokers was observed in the dACC among other brain regions [7, 8].

Here we compared two groups of adults with respect to MRS metabolites in the dACC—a group that smoked cigarettes and a nonsmoking control group. The hypothesis was that smoking-related hypoinflammation in the brain would manifest as elevated levels of NAA and Glu, but lower levels of Cr, Cho, and mI in participants who smoked relative to those who did not. Specifically, we posited that levels of each metabolite in the smoking group would vary from levels in nonsmoking participants in the direction opposite to that seen in known inflammatory disorders [12–15]. Although this approach risks oversimplification, we selected this straightforward strategy, reserving attempts to add complexity later if the data called for it. We additionally explored potential relationships between MRS metabolites in the dACC and clinical features of smoking—tobacco dependence, recent and lifetime exposure to smoking, cigarette craving, and psychological withdrawal.

## Participants and methods

### Experimental design

After overnight (~12 h) abstinence from smoking (Time 1), adults who smoked cigarettes daily provided self-report measures of cigarette craving and withdrawal and underwent MRS for the assay of neurometabolites in the dACC. The same morning, MRS scanning was repeated 25–55 min after participants smoked their first cigarette of the day (Time 2). A nonsmoking control group was scanned at comparable times (for test-retest comparison) but without smoking. Metabolite levels were analyzed for differences between the smoking and nonsmoking groups and between Time 2 and Time 1. Within the smoking group, associations between clinical features related to smoking and metabolite levels were tested.

### Participants

Participants for this study were recruited through online and print advertisements, as approved by the UCLA Institutional Review Board. During an intake session, all participants received a detailed explanation of the study procedures, gave written informed consent, and completed eligibility screening. Inclusion criteria were age 18–50 years and generally good health. Two groups were included: Individuals who smoke (“Smoking”) and individuals who do not smoke (“Nonsmoking”). For the smoking group, self-report of smoking at least 5 cigarettes per day for at least 1 year was required. Smoking status was verified by a cotinine concentration ≥200 ng/ml in urine (ACCUTEST 7 Nicolet® Urine and Saliva Screen, Jant Pharmacal Corp., Encino, CA) or ≥15 ng/ml in blood [25]. Nonsmoking participants denied smoking more than 5 cigarettes at any point in their lifetime, showed negative urinary cotinine, and registered breath CO < 10 ppm at intake. Exclusions for both groups were: positive urine test for substances other than nicotine or cannabis (use of cannabis up to seven times per week was allowed), consuming more than 10 alcoholic drinks a week in the preceding month, any current psychiatric disorder other than tobacco use disorder per the Mini International Neuropsychiatric Interview for DSM-5 [26, 27], history of brain injury linked to loss of consciousness of 30 min or more, and using forms of tobacco other than cigarettes more than three times per month.

### Verification of abstinence from alcohol and other drugs

Participants who met eligibility criteria returned on another day for further testing, including structural magnetic resonance imaging (MRI) and MRS scanning. Abstinence from cocaine, morphine, benzodiazepines, and amphetamines was verified with a five-panel urine drug test (Drugs of Abuse Test Insta-view®, Alfa Scientific Designs Inc., Poway, CA). Alcohol abstinence was verified using a breathalyzer (Alco-Sensor FST®, Intoximeters, Inc., St. Louis, MO). Recent abstinence from cannabis was verified with the Dräger DrugTest® 5000 saliva test (Dräger, Inc., Houston). Overnight (~12 h) abstinence from smoking was verified with the Micro + Smokerlyzer® breath CO monitor (Bedford Scientific Ltd., Maidstone, Kent, UK) with the criterion of CO in expired air <10 ppm or ≤50% the value at intake.

### Self-report measures

At intake, the Fagerström Test for Nicotine Dependence (FTND) [28] was administered to assess current tobacco dependence, and the Smoking History Questionnaire (SHQ) [29] was administered to assess recent tobacco exposure (cigarettes per day over the past 30 days) and lifetime tobacco exposure (pack-years). On the scanning day, participants in the smoking group completed the Craving and Psychological Withdrawal subscales of the Shiffman–Jarvik Withdrawal Scale [30].

### MR acquisition

Participants were scanned at 3 T on a Siemens Prisma with 32-channel phased-array head coil. High-resolution sagittal whole-brain T1-weighted structural MRI was acquired using a Magnetization-Prepared Rapid Gradient Echo (MPRAGE) pulse-sequence before each MRS scan that was used to position the dACC voxel and for offline MRS voxel tissue-content determination.

**Fig. 1:**
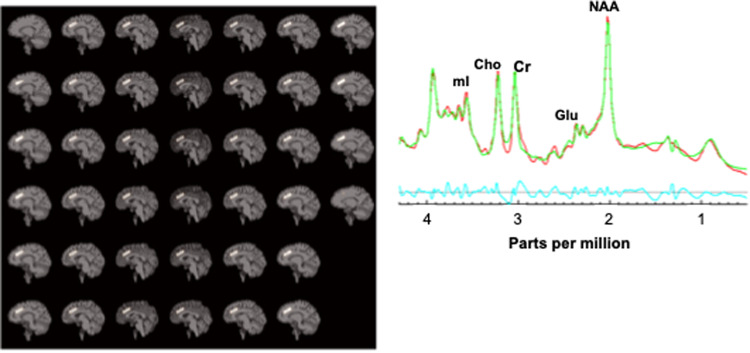
Acquisition and spectral fitting of single-voxel proton magnetic resonance spectroscopy (MRS). (left) Sagittal T1w-MRI skull-stripped sections of the brain of a representative study participant showing location of MRS (PRESS TR/TE = 1500/30 ms, 30 × 30 × 10 mm^3^, 400 excitations) acquisition voxel (white box) in midline dorsal anterior cingulate (dACC). (right) Sample dACC PRESS MR spectrum (plot of radiofrequency intensity *vs*. chemical shift) showing major neurometabolite resonances measured in this study. The red trace is the raw data, the green is the fit spectrum, and light blue is the residual (raw – fit) spectrum. NAA *N*-acetyl compounds, Glu glutamate, Cr creatine + phosphocreatine, Cho choline-compounds, mI myo-inositol.

Water-suppressed ^1^H MRS was acquired using single-voxel point-resolved spectroscopy (PRESS; Siemens svs_se pulse-sequence, repetition-time/echo-time = 1500/30 ms, 400 excitations, runtime 10:08 min). The acquisition voxel sampled midline (left+right) dACC, positioned approximately as in [31] (Fig. 1). The dACC voxel rested with its inferior face parallel to the dorsal corpus callosum. Left-right the voxel straddled the longitudinal fissure with its lateral faces bordering white matter. Its rostral face bordered the pregenual anterior cingulate cortex; its caudal face bordered the posterior middle cingulate cortex. Its axial-oblique dorsal face bordered the mesial superior frontal cortex. Voxel dimensions were 30 × 30 × 10 mm^3^ (left-right by anterior-posterior by inferior-superior) with position adjusted to maximize gray-matter content. An identical non-water-suppressed MRS voxel (8 excitations) was acquired immediately after the water-suppressed acquisition to enable water-referenced quantitation of metabolite levels. Possible head movement during MRS was checked for by comparing the brain position on rapid T1-weighted whole-brain scouts acquired before and after MRS.

### MRS post-processing

Tissue composition within each MRS voxel was determined as follows: The BET and FAST tools in FSL 5.0 (www.fmrib.ox.ac.uk/fsl/) were applied to the MPRAGE volume on which each MRS voxel was positioned. This procedure yielded whole-brain gray-matter, white-matter, and cerebrospinal fluid (CSF) native-space binary masks aligned with the MPRAGE. The tissue-content in the MRS voxels was calculated using these masks. MRS voxels with over 60% CSF were rejected. Each non-water-suppressed/water-suppressed MRS voxel pair was displayed on its corresponding MPRAGE volume (Fig. 1). The MRS scan was rejected either if the voxel was not centered in the dACC or if the two voxels were not co-aligned, as confirmed by <10% disparity in tissue-composition. Similarly, voxels were compared between the two measurements for each participant and were discarded in cases of mismatch.

MRS data from the 32 channels were combined for each water-suppressed and non-water-suppressed scan. Further processing was done with SVFit2016 [32], including time-domain filtering and non-linear least-squares spectral fitting to determine neurometabolite levels in each voxel. SVFit2016 uses the Levenberg–Marquardt implementation of the Gauss-Newton method to fit spectra in the frequency domain. The specific fitting routine is a modified version of MPFIT [33]. Fits for non-water-suppressed spectra used a model spectrum that included only a single water signal. Fits for water-suppressed spectra included models of spectra for lactate (Lac), *N*-acetylaspartate, *N*-acetylaspartylglutamate, Glu, Gln, γ-aminobutyric acid (GABA), creatine, phosphocreatine, Cho, mI, numerous low-level neurometabolites, residual water, lipids, and macromolecules. Model spectra were simulated in Versatile Simulation, Pulses, and Analysis software (VESPA) [34, 35]. Following fitting, the *N*-acetylaspartate and *N*-acetylaspartylglutamate signals were summed to total *N*-acetyl compounds (“NAA”). Similarly, creatine and phosphocreatine were summed to total creatine (“Cr”). Fit quality for all spectra was reviewed by two experts in the investigative team (JRA, JON). Non-water-suppressed spectra with excessive lipids, overly broad linewidth, distorted lineshape, or gross artifacts were rejected. Water-suppressed spectra were rejected for any of the foregoing reasons or for inadequate water-suppression or total creatine signal-to-noise ratio <5.

Metabolite levels were normalized to the unsuppressed water signal, corrected for voxel CSF-content, and expressed in Institutional Units (IU). To account for variations in gray-matter and white-matter content of the MRS voxels, metabolite levels were extrapolated to 100% gray-matter values. These “gray-matter extrapolated” values were the final endpoints for MRS data analysis.

### Statistical analysis

SPSS Version 28.0 was used for statistical analyses. Mean values of demographic and drug-use variables were compared between groups using independent *T*-tests. Data were screened for outliers using the method of quartiles [36]. Individual values of MRS NAA, Glu, Glx, Cr, Cho, or mI with Cramér-Rao lower bounds (CRLB) of >20% were excluded. Linear Mixed Model (LMM) analysis, which accounts for missing values, simultaneously evaluated the main effects of group (Smoking *vs*. Nonsmoking), sex (women *vs*. men), and repeated measurement (Time 2 *vs*. Time 1), and the smoking-by-sex interaction for each MRS metabolite level in the dACC, with age as a covariate. Sex was included as an independent variable based on a priori evidence of sex differences related to smoking [37–41] and of sex differences in metabolites irrespective of smoking [42–44]. The repeated measures testing assessed the reliability of the MRS scans and, in Smoking participants, detected any effects of breaking abstinence still evident 25–55 min after smoking. Histograms and normal Q-Q plots were inspected for each metabolite level to exclude appreciable deviation from normality. For the primary Smoking *vs*. Nonsmoking group comparisons of metabolite levels, the criterion for significance was set at *p* = 0.01 (0.05/5 metabolites) two-tailed to implement Bonferroni correction for multiple comparisons. All other tests were exploratory and therefore uncorrected. The sample size was chosen using the G*Power 3.1 ANOVA: Repeated measures module to target adequate power for pre-specified effect sizes of 0.53 for smoking, 1.01 for sex, and 0.4 for repeated measurement.

Although one prior MRS study about smoking [45] calculated metabolite levels relative to water, as in this study, several others [18, 22, 24] normalized to Cr. In animal studies, nicotine exposure has been associated with altered blood brain barrier (BBB) permeability via tight junction modulation [46] and with elevated water content and aquaporin-4 (AQP4) in spinal cord [47]. Such effects could conceivably alter the value of the denominator used in water quantitation of metabolites. This possibility renders normalizing to Cr a reasonable, perhaps even preferable, alternative for smoking MRS studies. Therefore, LMM was repeated for the MRS endpoints NAA/Cr, Glu/Cr, Cho/Cr, and mI/Cr. LMM was also repeated using any demographic and drug-use variables with significant between-group differences as covariates. The variables for which this was the case were education, mother’s education, and alcohol consumption (see below).

Relationships of metabolite levels with FTND score, cigarettes per day, pack-years, and the Shiffman–Jarvik subscales were also determined using Pearson correlation partialling age and sex. To test these associations, mean metabolite values of Time 1 and Time 2 were used. As noted in the Results below (except for mI/Cr), these values did not differ significantly from each other, and therefore could be collapsed.

All tests performed for Glu were repeated for Glx (see Supplemental Information). Values for GABA are not reported as some investigators do not consider GABA measured with conventional PRESS at 3 T to be reliable. Given the light level of smoking among some participants in the Smoking group (<10 cigarettes per day), all tests were repeated for a Heavier Smoking subsample (*N* = 29 participants who smoked ≥10 cigarettes per day; see Supplemental Information).

## Results

### Demographics and clinical characteristics; tissue composition of MRS voxels

Data that passed all quality-control criteria were obtained from 54 participants in the Smoking group and 37 in the Non-smoking group (Table 1). The groups did not differ significantly in age, sex, or days of cannabis use. On average, the Smoking sample had 7% fewer years of education (*p* = 0.045), 8% fewer years of mother’s education (a proxy for socioeconomic status; *p* = 0.038), and 126.5% more alcohol-drinking days (*p* = 0.009). Therefore, statistical tests were repeated including education, mother’s education, and alcohol consumption in the LMM model. A representative MR spectrum and voxel prescription are shown in Fig. 1. Group-mean CRLB values for major MRS neurometabolites are listed in Table 2. Mean voxel gray-matter, white-matter, and CSF content did not differ significantly between groups or between Time 2 and Time 1 (Table 2; all *p*s > 0.05).

**Table 1:** Sample characteristics and clinical measures (number or mean ± sd).

	Nonsmoking	Smoking	t/ χ^2^	*p*
*N*	37	54	--	--
Age (years)	31.9 ± 7.4	33.9 ± 7.8	1.23	0.225
male/female	18/19	25/29	0.05	0.825
Education (years)	15.2 ± 2.3	14.1 ± 2.6	2.02	**0.045**
Mother’s education (years)	15.2 ± 2.6	14.0 ± 2.3	2.13	**0.038**
FTND Total Score	0	4.2 ± 2.2	--	--
cigarettes/day	0	12.1 ± 5.2	--	--
pack-years	0	9.5 ± 7.2	--	--
baseline expired CO (ppm)	2.0 ± 0.9	13.0 ± 7.5	7.80	3x10^-14^
Alcohol use (days/month)	3.4 ± 3.7	7.7 ± 8.7	2.70	**0.005**
Cannabis use (days/month)	3.4 ± 9.0	5.7 ± 9.8	0.68	0.504

*FTND* Fagerström Test of Nicotine Dependence. Statistically significant *p* < 0.05 values are in bold (independent *T*-test).

**Table 2:** LMM analysis of dACC neurometabolite levels (mean ± sd).

	Nonsmoking		Smoking	
	Time 1	Time 2	Time 1	Time 2
*N*	35	19	49	41
gray matter (volume %)	51.5 ± 5.9	53.8 ± 6.2	52.5 ± 4.9	52.1 ± 5.6
white matter (volume %)	35.9 ± 6.9	31.6 ± 5.2	34.5 ± 6.2	34.9 ± 7.4
CSF (volume %)	11.9 ± 4.0	13.5 ± 4.1	12.9 ± 5.4	13.0 ± 4.6
NAA (IU)	21.3 ± 2.1	20.5 ± 2.2	**23.8** **±** **2.8**^**a**^	**23.4** **±** **3.0**^**a**^
CRLB (%SD)	4.2 ± 0.8	4.4 ± 0.9	4.7 ± 0.7	4.6 ± 0.8
Glu (IU)	17.5 ± 1.8	17.0 ± 2.2	**22.8** **±** **3.7**^**a**^	**22.5** **±** **2.7**^**a**^
CRLB (%SD)	6.7 ± 2.6	6.1 ± 1.2	6.4 ± 1.8	6.4 ± 1.3
Cr (IU)	17.4 ± 1.4	16.8 ± 1.5	**16.4** **±** **1.7**^**a**^	**16.1** **±** **1.8**^**a**^
CRLB (%SD)	1.9 ± 0.5	2.0 ± 0.4	1.9 ± 0.4	2.0 ± 0.4
Cho (IU)	4.5 ± 0.7	4.3 ± 0.5	**4.0** **±** **0.7**^**a**^	**3.9** **±** **0.6**^**a**^
CRLB (%SD)	2.0 ± 0.5	2.0 ± 0.4	2.1 ± 0.5	2.1 ± 0.5
mI (IU)	11.8 ± 2.7	12.4 ± 1.7	12.6 ± 2.6	12.8 ± 2.0
CRLB (%SD)	7.3 ± 3.3	6.1 ± 2.1	6.8 ± 2.2	6.4 ± 1.8
NAA/Cr	1.22 ± 0.13	1.22 ± 0.13	**1.46** **±** **0.13**^**a**^	**1.46** **±** **0.10**^**a**^
Glu/Cr	1.01 ± 0.13	1.01 ± 0.15	**1.39** **±** **0.20**^**a**^	**1.41** **±** **0.14**^**a**^
Cho/Cr	0.26 ± 0.03	0.25 ± 0.02	**0.25** **±** **0.03**^**b**^	**0.24** **±** **0.03**^**b**^
mI/Cr	0.68 ± 0.14	0.74 ± 0.11	**0.77** **±** **0.15**^**b**^	**0.80** **±** **0.15**^**bc**^

For gray matter, white matter, or CSF there were no significant within-sample differences between Time 2 and Time 1 means or between-sample differences between Smoking and Nonsmoking means on repeated-measures linear mixed-model (LMM) accounting for sex and age. For all metabolite levels, there were no significant within-sample differences between Time 2 and Time 1 on LMM accounting for sex and age. Statistically significant values are in bold (*p* < 0.01 on LMM). ^a^*p* < 0.001, ^b^*p* < 0.005 main effect of Smoking *vs*. non-smoking covarying age. ^c^*p* < 0.05 main effect of time.

*dACC* dorsal anterior cingulate cortex. *Time 1* after overnight abstinence. *Time 2* 25–55 min after the first cigarette of the morning (or corresponding times for Nonsmoking sample). *NAA* *N*-acetyl compounds. *Glu* glutamate. *Cr* creatine + phosphocreatine. *Cho* choline-compounds. *mI myo*-inositol. *IU* Institutional Units. *CRLB* Cramér-Rao lower bounds. *%SD* percent standard deviation.

### Metabolite levels: smoking status, sex, and repeated measurement (see Supplemental Information for Glx results)

**Fig. 2:**
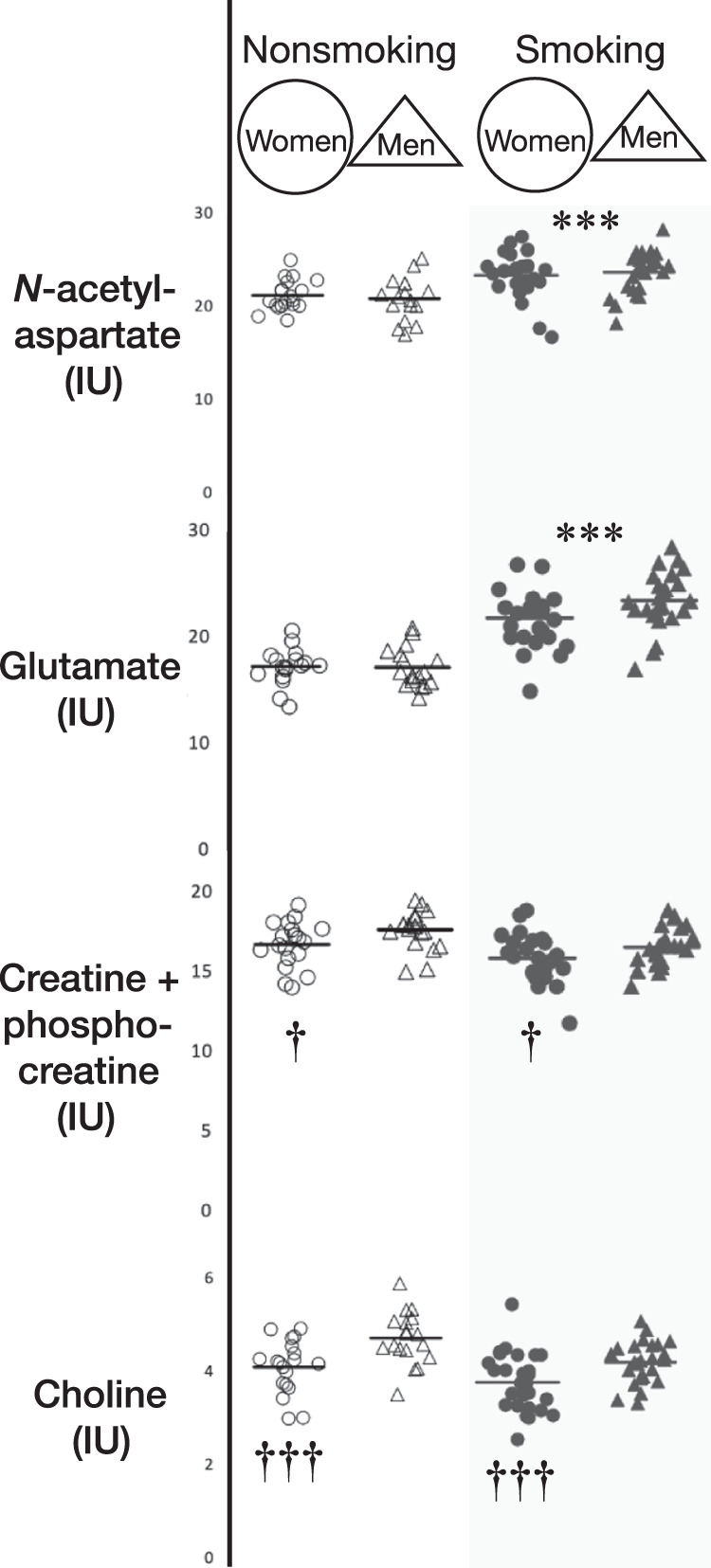
Smoking *vs*. non-smoking and sex group comparisons of neurometabolite levels in the dorsal anterior cingulate cortex. *N*-acetyl compounds were 11.7% higher in the Smoking than in the Non-smoking group (*p* < 0.001). Glutamate was 31.4% higher in the Smoking (combined male and female) than in the Non-smoking group (*p* < 0.001). Creatine + phosphocreatine was 5.5% lower in the Smoking than in the Non-smoking group (*p* < 0.001). Creatine + phosphocreatine was also 4.6% lower in women than in men (combined Smoking and Nonsmoking) independent of smoking status (*p* = 0.021). Choline compounds were 9.8% lower in the Smoking than in the Nonsmoking group (*p* < 0.001) and were 11.5% lower in women than in men (*p* < 0.001). Data are averages of Time 1 and Time 2 scans. Horizontal bars represent group means. All tests are repeated-measures linear mixed model covarying age. IU Institutional Units. ***p < 0.001 for the Smoking vs. Nonsmoking group (combined data from women and men); †*p* < 0.05, †††*p* < 0.001 for the female vs. male group (combined Smoking and Nonsmoking).

Significant main effects of group for NAA, Glu, Cr, and Cho, but not for mI, were found using LMM. For the Smoking group, NAA was 11.7% higher (F_1,137_ = 34.3, *p* < 0.001), Glu was 31.2% higher (F_1,132_ = 118.2, *p* < 0.001), Cr was 5.5% lower (F_1,136_ = 13.3, *p* < 0.001), and Cho was 9.8% lower (F_1,138_ = 25.1, *p* < 0.001) than for the Nonsmoking group (Table 2, Fig. 2). These effects were significant after Bonferroni-correction for multiple comparisons. There were significant main effects of sex for Cr and Cho. Thereby, for women *vs*. men (combined Smoking and Nonsmoking samples; Fig. 2), Cr was 4.6% lower (F_1,136_ = 5.4, *p* = 0.021) and Cho was 11.5% lower (F_1,138_ = 17.4, *p* < 0.001). The group-by-sex interaction was not significant for any metabolite (all *p*s > 0.05). There was no significant main effect of sampling time on any metabolite level (Table 2; all *p*s > 0.05). Therefore, averages of Time 1 and Time 2 metabolite levels from each participant were used when computing group differences and for correlations with FTND, cigarettes per day, and pack years.

Findings for Glx were highly similar to those for Glu (Supplemental Information); findings for the Heavier Smoking subsample were highly similar to those for the entire Smoking group (Supplemental Information). All findings retained statistical significance after including education, mother’s education, and alcohol consumption in LMM. Effects of alcohol consumption were (F_1,86_ = 0.1, *p* = 0.735) for NAA, (F_1,83_ = 0.5, *p* = 0.470) for Glu, (F_1,85_ = 0.1, *p* = 0.874) for Cr, (F_1,86_ = 0.4, *p* = 0.508) for Cho, and (F_1,86_ = 1.5, *p* = 0.225) for mI.

### Metabolite ratios to Cr: smoking status, sex, and repeated measurement (see Supplemental Information for Glx/Cr results)

Significant main effects of the group for NAA/Cr, Glu/Cr, Cho/Cr, and mI/Cr were found using LMM. For the Smoking group, NAA/Cr was 19.7% higher (F_1,135_ = 154.5, *p* < 0.001), Glu/Cr was 38.6% higher (F_1,132_ = 189.1, *p* < 0.001), Cho/Cr was 7.7% lower (F_1,136_ = 8.9, *p* = 0.003), and mI/Cr was 12.9% higher (F_1,136_ = 9.2, p = 0.003) than for the Nonsmoking group (Table 2). There were significant main effects of sex for NAA/Cr and Cho/Cr. Thereby, for women vs. men (combined Smoking and Nonsmoking groups; Fig. 2), NAA/Cr was 4.5% higher (F_1,135_ = 8.0, *p* = 0.005) and Cho/Cr was 8.3% lower (F_1,136_ = 10.3, *p* = 0.002). The group-by-sex interaction was not significant for any metabolite (all *p*s > 0.05). mI/Cr was 6.8% higher (F_1,136_ = 4.0, *p* = 0.048) at Time 2 than at Time 1; otherwise, there was no significant main effect of sampling time on any metabolite ratio (Table 2; all *p*s > 0.05). Thus, averages of Time 1 and Time 2 metabolite levels from each participant were used when computing group differences and for correlations with FTND, cigarettes per day, and pack years; but for mI/Cr only, separate correlations were computed for Time 1 and Time 2.

Findings for Glx/Cr were highly similar to those for Glu/Cr (Supplemental Information); findings for the Heavier Smoking subsample were highly similar to those for the entire Smoking group (Supplemental Information).

### Associations of metabolite levels with smoking variables (also see Supplemental Information)

Within the Smoking sample, Glu was negatively correlated with FTND (*r* = −0.34, *p* = 0.016) and Cr was negatively correlated with FTND (*r* = −0.44, *p* = 0.001) and with pack-years (*r* = −0.33, *p* = 0.017) (Table 3, Fig. S1 Supplemental Information). No dACC metabolite was significantly associated with the Shiffman–Jarvik Craving or Psychological Withdrawal subscale scores (all *p*s > 0.05). NAA/Cr, Glu/Cr, and Cho/Cr (means of Time 1 and Time 2) were not significantly associated with any smoking variable (all ps >0.05); mI/Cr did not correlate significantly with any of these variables at Time 1 or at Time 2.

**Table 3:** Associations of dACC MRS neurometabolite levels with tobacco dependence in Smoking group.

	FTND			CPD			Pack-Years		
	df	r	p	df	r	p	df	r	p
NAA	50	-0.26	0.059	50	-0.12	0.397	50	-0.18	0.207
Glu	**49**	**-0.34**	**0.016**	49	-0.21	0.148	49	-0.13	0.368
Cr	**49**	**-0.44**	**0.001**	49	-0.26	0.069	**49**	**-0.33**	**0.017**
Cho	50	-0.17	0.236	50	-0.07	0.602	50	-0.10	0.478
mI	50	-0.04	0.803	50	-0.10	0.485	50	-0.08	0.547

Results are for Pearson correlation partialling sex and age within the Smoking sample. Statistically significant *p* < 0.05 values are in bold.

dACC dorsal anterior cingulate cortex. *MRS* magnetic resonance spectroscopy. *FTND* Fagerström Test for Nicotine Dependence. *CPD* cigarettes per day. *NAA N*-acetyl compounds. *Glu* glutamate. *Cr* creatine + phosphocreatine. *Cho* choline-compounds. *mI myo*-inositol.

## Discussion

In this study of neurometabolites in the dorsal anterior cingulate cortex (dACC), NAA and Glu were higher and Cr and Cho were lower in smoking than nonsmoking research participants. Glu correlated negatively with tobacco dependence and Cr correlated negatively with both tobacco dependence and lifetime smoking history. Independent of smoking status, Cr and Cho were lower in women than men. These findings are largely consistent with a hypoinflammatory state in the dACC of people who smoke, although alternative interpretations remain viable.

Multiple putative MRS markers of neuroinflammation differed between the Smoking and Nonsmoking groups, consistent with previous evidence that people who smoke have below-control levels of inflammatory markers in the dACC [7, 8]. The present findings encompassed higher NAA and Glu and lower Cr and Cho in the Smoking group. All of these findings are consistent with the downregulation of inflammatory mediators and are in the direction opposite to that seen in many disorders that feature *increased* neuroinflammation [13] (further discussion in Supplemental Information). Similar findings were obtained for the metabolite ratios NAA/Cr, Glu/Cr, and Cho/Cr, implying that these results were not solely due to smoking-induced changes in the BBB, AQ4, or tissue water content [46, 47]. One way that smoking may induce a hypoinflammatory state in the brain is through the agonist action of nicotine (and other ligands contained in tobacco smoke) at α-7 nicotinic acetylcholine receptors (nAChRs), which are expressed in microglia [48]. By acting on α-7 nAChRs, nicotine attenuates the proinflammatory actions of microglia, including proton currents and the microglial morphological transition [49], modulating microglia towards a neuroprotective, as opposed to their better-known neuroinflammatory role [50]. The MRS findings observed here may reflect effects downstream from such actions on microglia. One should, however, note that the interaction of smoking with the immune system is complex and includes both pro-inflammatory and suppressive inflammatory effects [51, 52].

Low NAA is attested in many inflammatory conditions [13], where it is thought to reflect decreased neuronal function and viability. Additionally, in an in vitro study of human astroglial cells [53], NAA exerted direct anti-inflammatory actions, decreasing COX-2 protein and activating NF-κB. Elevated NAA in the Smoking group here may represent elevated neuron density or metabolic hyperactivity, and may contribute to hypoinflammation in the dACC.

Glu (or Glx or Glx/Cr) in the dACC or proximal cortices in smoking has been the focus of prior studies. Higher dACC Glu in the Smoking than the Nonsmoking group in this study is consistent with previous findings of elevated glutamatergic metabolites in dACC [22] or neighboring posterior middle cingulate [23] in people who smoke, although this finding is at variance with lower Glu in smoking participants in one report [45]. Below-control levels of Glu occur in certain inflammation-linked disorders [13] where, like low NAA, they are seen as a sign of neuronal function loss. Accordingly, we interpret *elevated* Glu in the Smoking group here as a further indication of a *hypoinflammatory* state. Evidence to support this notion includes the observation that nicotine changes the functioning and morphology of astrocytes—cells integral to Glu metabolism—without inducing their inflammatory reactive transition [54]. Further astrocytic effects of nicotine include reduction of Glu uptake through GLT1 transporters and slower conversion of Glu to Gln by astrocytic glutamine synthetase [55, 56]. Such effects increase synaptic Glu transiently, but would not likely sustain chronically elevated Glu in people who smoke. More fundamentally, such inhibitory effects would probably not lead to excess Glu as they simultaneously impede the production of astrocytic Gln that is shuttled back to the neuron where it ultimately restores Glu levels.

Although Glu was higher in the Smoking group than in the Nonsmoking group, Glu correlated negatively with nicotine dependence (FTND score) within the Smoking group. This correlation was stronger in the Heavier Smoking subsample (Supplemental Information). There are ways one might explain this counterintuitive negative correlation. It may, for example, represent a homeostatic response to keep dACC Glu levels from rising too high or, alternatively, elevated Glu may reflect a risk factor for tobacco use disorder. But, since similar correlations were not observed for Glx, Glu/Cr, or Glx/Cr, this finding should be viewed with caution.

That both NAA and Glu were higher in the Smoking group may also seem counterintuitive since Glu is neurotoxic and NAA is a marker of neuronal integrity. This apparent contradiction is resolved when one considers the distribution of Glu within brain tissue (see [57]). Most brain Glu occurs in three environments. The greatest portion of Glu is in the cytoplasm of neurons and glia; lesser fractions are found in synaptic vesicles and in glial and neuronal mitochondria [58, 59]. Due to the neurotoxicity and rapid clearance of extracellular Glu, rather little Glu exists in the synaptic cleft or other extracellular spaces in healthy brains [59]. Moreover, experiments imply that ~25% of brain Glu is “invisible” to MRS [58, 60–63], even at our TE of 30 ms [57]. Moreover, the (potentially excitotoxic) vesicular Glu is thought to belong to this invisible fraction [58, 63]. Thus, the present observed effects of smoking on Glu are unlikely to represent elevated synaptic or other extracellular Glu. Rather, they more likely reflect an adaptive shift in intracellular metabolism resulting in the expansion of the overall tissue pool of Glu, or net transfer of Glu from the invisible to the “visible” cytoplasmic environment. These mechanisms do not imply increased excitotoxicity and, hence, are quite compatible with elevated NAA.

Elevated Cr and Cho occur in inflammatory disorders [13], suggesting that low Cr and Cho in the Smoking sample here are further signs of hypoinflammation. Cr in the Smoking group additionally was lower for greater tobacco dependence and lifetime exposure. Regarding high Cho, we recently presented further evidence that it is a marker of neuroinflammation. In a placebo-controlled clinical trial, the anti-inflammatory drug ibudilast lowered Cho in white matter in patients with alcohol use disorder [64]. Cho also correlated with the volume of dilated perivascular spaces, which are associated with neuroinflammation, in white matter in Parkinson’s Disease [65]. In sum, similar to prior PET evidence [7, 8], present MRS findings are consonant with dACC hypoinflammation in people who smoke.

Although hypoinflammation is a parsimonious interpretation of the present findings, alternative explanations and effects are not excluded. Various physiological roles have been posited for these metabolites, which in principle, may be influenced by smoking. NAA, for example, may reflect mitochondrial activity [66], contribute to myelin synthesis [67], and be a storage form of acetyl-CoA [68]. Glu is well known as a neurotransmitter [69] and a marker of Krebs Cycle activity [70]. Cr, in addition to its role in brain energetics buffering glycolysis and oxidative phosphorylation [71], may modulate NMDA receptors [72] and neuronal discharge [73, 74] and act as a neuroprotectant [75]. Cho is frequently cited as a marker of cell membrane turnover [71], and Cho effects could also result from energy failure, membrane degradation, angiopathy, and edema [76]. All four metabolites additionally serve as osmolytes [77]; hence their levels may have been altered by the effects of smoking on water homeostasis, although findings of the present study differed little between metabolites normed to water and metabolites normed to Cr.

Some investigators are skeptical that MRS metabolites are biomarkers of neuroinflammation at all. Counterarguments include insufficient histopathological support for neuroinflammatory interpretations of MRS findings [76]. For example, mI is often claimed to be a glial marker based on a study of juvenile rat-brain extracts whose findings may not extrapolate to humans or adults, while another study found no difference in mI between human neuronal and glioma extracts. In simian immunodeficiency virus (SIV), Cr correlated neither with the astrocyte marker glial fibrillary acidic protein (GFAP) nor with the microglial marker ionized calcium binding adaptor molecule 1 (although Cr did correlate modestly with gliosis and NAA/Cr did correlate negatively with inflammatory elements such as perivascular histiocytic infiltrates, multinucleated giant cells, and CD14 + CD16+ monocytes in the brain). Thus, MRS markers alone are insufficient to identify neuroinflammation, and multimodal approaches are called for.

Respecting these critiques, alternative interpretations of the present MRS results are possible, but the present findings do accord with prior PET studies of smoking [7, 8]. Moreover, not all interpretations of MRS metabolites are mutually exclusive. In inflammation, activated astrocytes operate at lower energy efficiency and are diverted from their normal roles in brain energy production. Energy failure therefore could result from inflammation. Similarly, an inflammatory response can include membrane degradation and edema. The existence of alternatives does not rule-out the hypoinflammatory explanation of our findings, but the concept of MRS metabolites as markers of neuroinflammation remains in need of better support through future histopathological studies.

The level of mI was *not* lower in the Smoking group than in the Non-smoking group despite evidence that high mI is a marker of neuroinflammation [13]. A possible explanation of this negative finding is that, as stated by others [15], each neuroimaging marker of inflammation marks a different stage or aspect of inflammation, and which markers do so vary by disease. Hence, not all markers need to manifest in every condition. The only metabolite marker in the present study to show a significant elevation at Time 2 compared to Time 1 was mI/Cr; this may be further evidence of a separate role for mI relative to the other metabolites. In addition, mI may be an insensitive marker of hypoinflammation in smoking. Group differences in mI findings may have been absent in our study because mI is more difficult to quantitate at an echo-time of 30 ms, which was used here, than at shorter echo-times.

In contrast to our MRS findings, a recent study found lower NAA, Glu, Cr, and mI in participants who smoked as compared with a nonsmoking sample [45]. Although the Cr finding agrees with that observed here, the present study had no mI finding and its NAA and Glu findings were in the opposite direction. Methodological differences, particularly the prescription of the MRS voxels sampled, may have contributed to the discrepancies. Faulkner et al. sampled the right pregenual anterior cingulate cortex, which was lateral and anterior to our voxel in midline dACC. Also, their voxel contained ~50% white matter vs. 31–36% white matter in the present study. In addition, participants in the present study were on average ~10 years older, included more females, and had substantially greater lifetime smoking exposure than those studied by Faulkner et al. [45]. Our Smoking participants were tested in the morning after confirming overnight abstinence from smoking, but length of abstinence and time of scan are not reported in [45]. Finally, it is possible that Faulkner et al. obtained different mI findings because they used a shorter echo-time (8.5 ms) than in the present study.

Cr and Cho were lower in women than in men in the present study, findings that may also relate to neuroinflammation. Sex differences in neuroinflammation are widely reported [78, 79]; estradiol [80, 81] and progesterone [82, 83] have reduced neuroinflammation in preclinical studies. Given that women have higher estradiol and progesterone than men and that estrogen and progesterone help suppress neuroinflammatory responses, it is thus unsurprising that Cr and Cho were lower in women than in men.

With the exception of mI/Cr, metabolite levels and ratios did not differ significantly between Time 2 and Time 1 for either group. For the Nonsmoking group, this implies good scan-rescan reliability of the MRS measures. For the Smoking group, it does additionally suggest that the MRS findings are due to chronic, rather than acute, exposure to tobacco, as was concluded for the above-mentioned TSPO PET findings [7, 8], which were essentially the same after overnight abstinence and 15 min after smoking to satiety.

The fact that metabolite levels showed no significant associations with craving or psychological withdrawal was unexpected, given reports of higher Glx during smoking satiety than abstinence [22, 24, 84], and of higher dACC Glu accompanying greater engagement of the default mode network [18]. Additional studies matching the time-course of dACC Glu measurements with that of withdrawal and relief with smoking may clarify this issue.

There are limitations to our study. Although all participants in the Smoking group reported and provided biological evidence of daily smoking, the level of smoking in some of the samples was relatively light. Results, however, were highly similar for a Heavier Smoking subsample (Supplemental Information) as for the entire Smoking group. Our MRS voxel contained 19–52% white matter, but correction was implemented for gray-matter/white-matter content. MRS measurements were acquired in one region only, the dACC. Future studies may explore multi-voxel MRS methods for simultaneous sampling of multiple brain regions implicated in smoking-related states. In addition, sex differences observed here suggest that circulating hormones may affect the findings. Our study also included no circulatory markers of inflammation, e.g., C-reactive protein.

We report findings for Glu. MR spectra were acquired at 3 T using PRESS and 30 ms echo-time. Some investigators feel that Glu is poorly segregated from Gln under these conditions and maintain, therefore, that only Glx (Glu+Gln) should be reported (e.g., [23]). Other researchers have found it acceptable to report Glu under these conditions [85–88, and many more] and have done so in highly rigorous studies [89–91]. This decision is supported by the fact that spectral fitting programs, such as LCModel or the SVFit package used here, routinely return low CRLBs (i.e., good fits) for Glu under these conditions, especially when the data have been carefully quality controlled, as in our study. We opted to report both Glu and Glx (Supplemental Information) and found results highly similar for the two.

Strengths of the study included biological confirmation of smoking or nonsmoking status, overnight abstinence, and non-use of other drugs of abuse; rigorous quality control of all MR spectra; use of both water-referenced metabolite levels and ratios to Cr; and analysis of Glx to corroborate Glu results (see Supplemental Information). Weighing these strengths and limitations, we conclude that the present study is consistent with prior PET evidence of hypoinflammation in the dACC in tobacco use disorder. Because inflammation is a critical component of normal tissue repair and defense against infection, smoking may render the brain more susceptible to insult. A further alternative that we express with reservations but mention for balance, is that smoking may be protective against certain conditions. For example, one study showed that current smoking was associated with less severe inflammatory consequences of COVID-19 [92]. In the same study, however, the risk of severe COVID-19, hospitalization, ICU admission, and death were higher for individuals who had smoked in the past than for those who had never smoked. In addition, all-cause mortality was higher for individuals who endorsed current smoking than for those who never smoked. Smoking has also been shown to induce remission of the inflammatory condition ulcerative colitis [93].

### Supplementary information

Supplemental material

Spplemental Figure S1

## Data Availability

All self-report, toxicology, and summary MRS data reported in this manuscript are publicly available from the Open Science Framework web site under project title, “Smoking, tobacco dependence, and neurometabolites in the dorsal anterior cingulate cortex” (https://osf.io/bnqsv).
