# Supplementary material for: Smoking, tobacco dependence, and neurometabolites in the dorsal anterior cingulate cortex
Source: Mol Psychiatry. 2023 Sep 25;28(11):4756–65. doi: 10.1038/s41380-023-02247-0 (PMC10914613; doi:10.1038/s41380-023-02247-0)
Supplement: Supplementary file 1 — Supplemental material [file 41380_2023_2247_MOESM1_ESM.doc]

**SUPPLEMENTAL INFORMATION**

**SUPPLEMENTAL RESULTS**

**Plots of associations of metabolite levels with smoking variables**

Fig. S1 shows plots of significant associations described in the main text between smoking variables and metabolite levels within the Smoking group, in particular the plots for FTND negatively correlated with Glu (*r* = -0.33, *p* = 0.017), for Cr negatively correlated with FTND (*r* = -0.44, *p* = 0.001), and for Cr negatively correlated with pack-years (*r* = -0.33, *p* = 0.017).

**dACC Glx Levels and Glx/Cr Ratios**

Linear mixed model (LMM) revealed that Glx was 13.1% higher in the Smoking than the Nonsmoking group (F_1,133_ = 24.5, *p* <0.001) and 14.1% higher in the Heavier Smoking subsample than the Nonsmoking group (F_1,92_ = 18.7, *p* <0.001). For women *vs.* men, Glx was 7.6% lower (F_1,133_ = 8.0, *p* = 0.005) for the combined Smoking and Nonsmoking groups and 1.4% lower (F_1,92_ = 4.6, *p* = 0.035) for the combined Heavier Smoking subsample and Nonsmoking group. Within the Smoking group, a negative relationship was found for Glx with FTND (*r* = -0.32, *p* = 0.023). Within the Heavier Smoking subsample, that relationship was stronger (*r* = -0.54, *p* = 0.004). Glx levels did not differ significantly between Time 2 and Time 1 scans for the Smoking group, the Nonsmoking group, or the Heavier Smoking subsample. Thus, Glx results scarcely differed from Glu results. LMM revealed that Glx/Cr was 20% higher in the Smoking than the Nonsmoking group (F_1,131_ = 55.7, *p* <0.001) and also 20% higher in the Heavier Smoking subsample than the Nonsmoking group (F_1,91_ = 38.2, *p* <0.001). No significant relationship of Glx/Cr was found with any smoking variables within the Smoking group or Heavier Smoking subsample. Glx/Cr levels did not differ significantly between Time 2 and Time 1 scans for the Smoking group, the Nonsmoking group, or the Heavier Smoking subsample. Thus, Glx/Cr results were similar to those for Glu and Glu/Cr.

**dACC MRS Results for Heavier Smoking Subsample**

**Demographics and Clinical Characteristics**

The Heavier Smoking subsample of the Smoking group comprised 29 participants (Table S1). The Heavier Smoking subsample had 7.8% fewer years of education (*p* = 0.036) than the Nonsmoking group, but the two did not differ significantly in years of mother’s education, days of drinking alcohol, or cannabis use from the Nonsmoking group. The major findings described below remained significant after education was added to the statistical model.

**MRS Voxel Tissue Composition**

Voxel gray-matter, white-matter, or CSF content did not differ significantly between the Heavier Smoking subsample and the Nonsmoking group (Table S2). Nor did Time 2 voxel gray-matter content differ significantly from Time 1 values for the Heavier Smoking subsample (Table S2).

**dACC MRS Metabolite Levels: Sampling Time, Smoking Status, and Sex**

For the Heavier Smoking subsample *vs.* the Nonsmoking group (Table S2), LMM found that NAA was 12.8% higher (F_1,94_ = 34.6, *p* <0.001), Glu was 31.7% higher (F_1,90_ = 87.4, *p* < 0.001), Cr was 5.8% lower (F_1,94_ = 10.9, *p* <0.001), and Cho was 9.1% lower (F_1,95_ = 25.6, *p* <0.001). For women *vs.* men (combined Heavier Smoking subsample and Nonsmoking group), LMM revealed that Cr was 4.6% lower (F_1,94_ = 4.7, *p* = 0.033) and Cho was 13.3% lower (F_1,95_ = 21.1, *p* <0.001). The smoking-by-sex interaction was not significant for any metabolite when analyzing the Heavier Smoking subsample. There were no significant differences between mean Time 2 and Time 1 levels for any metabolite within the Heavier Smoking subsample (Table S2). Therefore, in further analyses, Time 1 and Time 2 metabolite values were collapsed. Thus, findings for Heavier Smoking participants did not differ noticeably from those for the overall Smoking group.

**dACC MRS Metabolite Levels: Associations With Smoking Variables**

Within the Heavier Smoking subsample (Table S3), there was a negative relationship of Glu with FTND (*r* = -0.54, *p* = 0.004). There were also negative relationships for Cr with FTND (*r* = -0.58, *p* = 0.002), cigarettes per day (*r* = -0.44, *p* = 0.024), and pack-years (*r* = -0.49, *p* = 0.012). Thus, there were signs of decreasing Glu and Cr for increasing nicotine dependence, amount of recent cigarette smoking, or amount of chronic smoking exposure. In contrast, no dACC metabolite had a significant relationship with the Shiffman-Jarvik Craving or Psychological Withdrawal subscale scores.

**SUPPLEMENTAL DISCUSSION**

**Putative MRS Markers of Neuroinflammation in dACC**

Here we present additional evidence from the literature for the view that MRS metabolite levels mark tissue inflammation. The abnormalities in MRS metabolites seen in many neuroinflammatory disorders are nicely tabulated in [1], where sources for this discussion can also be found. Below-normal NAA is attested in multiple sclerosis (MS) [2-4], HIV [5-8], hepatitis C (Hep C) [9-11], progressive multifocal leukoencephalopathy (PML) [12-14], cytomegalovirus [15], chronic regional pain syndrome [16], and fibromyalgia [17], among other inflammatory conditions. NAA concentration is widely considered to be proportional to neuronal mass or metabolic activity [18], hence, lower NAA is taken to indicate loss of function in inflamed nervous tissue. Further, one study [19] found evidence for direct anti-inflammatory actions of NAA. Elevated NAA in our Smoking group may represent (possibly pathological) high neuronal density or metabolic hyperactivity and may contribute to a hypoinflammation in the dACC.

Low Glu is found in the inflammation-linked disorders HIV [7,20,21], PML [1], and spinal cord injury [22]. It is interpreted, similarly to low NAA, as a sign of loss of neuronal function. Thus, we take the *high* levels of Glu in our present Smoking sample as a sign of hypoinflammation.

Note that *above*-normal Glu is seen in inflammatory conditions such as MS [4], Hep C [9,23], and fibromyalgia [24,25]. High Glu typically occurs in cases of acute pain, discomfort, or fulminating lesions. It is thought to accompany (possibly excitotoxic) neuronal hyperactivity that promotes hypersensitivity and hyperalgesia. In the context of smoking, as in the present study, two endpoints that might correspond to pain and discomfort are the craving and withdrawal scores. These did not correlate significantly with dACC Glu in our study or in a prior [26] study. That could be one argument that elevated Glu in our Smoking group does not imply acute inflammation. In particular, Glu levels at Time 1 (when craving and withdrawal were rather high) differed little from those at Time 1 (when craving and withdrawal were much lower). We therefore remain by the interpretation that high Glu in our Smoking participants indicates a hypoinflammatory state. Preclinical evidence hints at how elevated Glu might arise in the absence of inflammation. Nicotine can change the functioning and morphology of astrocytes—cells that are key in regulating Glu levels [27] -- without inducing the astrocytic inflammatory reactive transition [28]. Effects of nicotine on astrocytes include decreasing Glu uptake *via* astrocytic transporters and reducing astrocytic conversion of Glu to Gln *via* glutamine synthetase [29,30]. Such effects might contribute to elevated dACC Glu in people who smoke.

Elevated Cr has been found in the inflammatory disorders MS [1,31], HIV [1], Hep C [1], and spinal cord injury [22]. Thus, low Cr in our Smoking sample may be a further sign of hypoinflammation. Above-normal Cho is found in MS [31-33], HIV [34-37], Hep C [9,11,38,39], PML [12-14], spinal cord injury [22,40], temporo-mandibular disorder [1], and chronic fatigue syndrome (CFS) [41]. Hence, we take low Cho in smoking as another sign of hypoinflammation in dACC. In sum, similar to prior PET evidence [42,43], all four components of our first major finding are in harmony with hypoinflammation in dACC in smoking.

Elevated mI has been observed in several inflammatory conditions, including MS [44-46], HIV [34,47,48], Hep C [9], PML [12], cytomegalovirus [15], chronic regional pain syndrome [1], and spinal cord injury [17]. As discussed in the main text, contrary to our hypothesis, mI was *not* lower in the Smoking sample, even though evidence for high mI as a marker of neuroinflammation is strong. A possible explanation for the absence of an mI finding is that inflammation varies by disease and by stage within each disease [49], hence, not every neuroimaging marker of inflammation need manifest in every stage and for every pathological condition. Alternatively, mI may have been poorly quantifiable at our echo-time (TE) of 30 ms, as opposed to a TE of 20 ms or less.

**SUPPLEMENTAL REFERENCES**

1 Chang L, Munsaka SM, Kraft-Terry S, Ernst T. Magnetic resonance spectroscopy to assess neuroinflammation and neuropathic pain. J Neuroimmune Pharmacol. 2013;8(3):576-93.

2 Kirov II, Tal A, Babb JS, Herbert J, Gonen O. Serial proton MR spectroscopy of gray and white matter in relapsing-remitting MS. Neurology. 2013;80(1):39-46.

3 Rigotti D, Inglese M, Kirov I, Gorynski E, Perry N, Babb J, et al. Two-year serial whole-brain N-acetyl-L-aspartate in patients with relapsing-remitting multiple sclerosis. Neurology. 2012;78(18):1383-89.

4 Srinivasan R, Sailasuta N, Hurd R, Nelson S, Pelletier D. Evidence of elevated glutamate in multiple sclerosis using magnetic resonance spectroscopy at 3 T. Brain. 2005;128(5):1016-25.

5 Chang L, Cloak C, Yakupov R, Ernst T. Combined and independent effects of chronic marijuana use and HIV on brain metabolites. J Neuroimmune Pharmacol. 2006;1(1):65-76.

6 Chang L, Ernst T, Leonido-Yee M, Walot I, Singer E. Cerebral metabolite abnormalities correlate with clinical severity of HIV-1 cognitive motor complex. Neurology. 1999;52(1):100-00.

7 Mohamed MA, Barker PB, Skolasky RL, Selnes OA, Moxley RT, Pomper MG, et al. Brain metabolism and cognitive impairment in HIV infection: a 3-T magnetic resonance spectroscopy study. Magn Reson Imaging. 2010;28(9):1251-57.

8 Schweinsburg BC, Taylor MJ, Alhassoon OM, Gonzalez R, Brown GG, Ellis RJ, et al. Brain mitochondrial injury in human immunodeficiency virus-seropositive (HIV+) individuals taking nucleoside reverse transcriptase inhibitors. J Neurovirol. 2005;11(4):356-64.

9 Grover V, Pavese N, Koh SB, Wylezinska M, Saxby B, Gerhard A, et al. Cerebral microglial activation in patients with hepatitis C: in vivo evidence of neuroinflammation. J Viral Hepat. 2012;19(2):e89-e96.

10 McAndrews MP, Farcnik K, Carlen P, Damyanovich A, Mrkonjic M, Jones S, et al. Prevalence and significance of neurocognitive dysfunction in hepatitis C in the absence of correlated risk factors. Hepatology. 2005;41(4):801-08.

11 Weissenborn K, Krause J, Bokemeyer M, Hecker H, Schüler A, Ennen JC, et al. Hepatitis C virus infection affects the brain—evidence from psychometric studies and magnetic resonance spectroscopy. J Hepatol. 2004;41(5):845-51.

12 Chang L, Ernst T, Tornatore C, Aronow H, Melchor R, Walot I, et al. Metabolite abnormalities in progressive rnultifocal leukoencephalopathy by proton magnetic resonance spectroscopy. Neurology. 1997;48(4):836-44.

13 Iranzo A, Moreno A, Pujol J, Martí-Fàbregas J, Domingo P, Molet J, et al. Proton magnetic resonance spectroscopy pattern of progressive multifocal leukoencephalopathy in AIDS. J Neurol Neurosurg Psychiatry. 1999;66(4):520-23.

14 Yoon JH, Bang OY, Kim HS. Progressive multifocal leukoencephalopathy in AIDS: proton MR spectroscopy patterns of asynchronous lesions confirmed by serial diffusion-weighted imaging and apparent diffusion coefficient mapping. J Clin Neurol. 2007;3(4):200-03.

15 Van der Voorn J, Pouwels P, Vermeulen R, Barkhof F, Van der Knaap M. Quantitative MR imaging and spectroscopy in congenital cytomegalovirus infection and periventricular leukomalacia suggests a comparable neuropathological substrate of the cerebral white matter lesions. Neuropediatrics. 2009;40(04):168-73.

16 Fukui S, Matsuno M, Inubushi T, Nosaka S. N-Acetylaspartate concentrations in the thalami of neuropathic pain patients and healthy comparison subjects measured with 1H-MRS. Magn Reson Imaging. 2006;24(1):75-79.

17 Pattany PM, Yezierski RP, Widerström-Noga EG, Bowen BC, Martinez-Arizala A, Garcia BR, et al. Proton magnetic resonance spectroscopy of the thalamus in patients with chronic neuropathic pain after spinal cord injury. Am J Neuroradiol. 2002;23(6):901-05.

18 Rae CD. A guide to the metabolic pathways and function of metabolites observed in human brain 1H magnetic resonance spectra. Neurochem Res. 2014;39(1):1-36.

19 Rael LT, Thomas GW, Bar-Or R, Craun ML, Bar-Or D. An anti-inflammatory role for N-acetyl aspartate in stimulated human astroglial cells. Biochem Biophys Res Commun. 2004;319(3):847-53.

20 Chang L, Ernst T, Witt MD, Ames N, Gaiefsky M, Miller E. Relationships among brain metabolites, cognitive function, and viral loads in antiretroviral-naıve HIV patients. Neuroimage. 2002;17(3):1638-48.

21 Ernst T, Jiang CS, Nakama H, Buchthal S, Chang L. Lower brain glutamate is associated with cognitive deficits in HIV patients: A new mechanism for HIV‐associated neurocognitive disorder. J Magn Reson Imaging. 2010;32(5):1045-53.

22 Widerström-Noga E, Pattany PM, Cruz-Almeida Y, Felix ER, Perez S, Cardenas DD, et al. Metabolite concentrations in the anterior cingulate cortex predict high neuropathic pain impact after spinal cord injury. Pain. 2013;154(2):204-12.

23 Forton DM, Allsop JM, Main J, Foster GR, Thomas HC, Taylor-Robinson SD. Evidence for a cerebral effect of the hepatitis C virus. Lancet. 2001;358(9275):38-39.

24 Harris RE, Clauw DJ. Imaging central neurochemical alterations in chronic pain with proton magnetic resonance spectroscopy. Neurosci Lett. 2012;520(2):192-96.

25 Harris RE, Sundgren PC, Pang Y, Hsu M, Petrou M, Kim SH, et al. Dynamic levels of glutamate within the insula are associated with improvements in multiple pain domains in fibromyalgia. Arthritis Rheumatol. 2008;58(3):903-07.

26 Schulte M, Goudriaan A, Kaag A, Kooi D, Van Den Brink W, Wiers R, et al. The effect of N-acetylcysteine on brain glutamate and gamma-aminobutyric acid concentrations and on smoking cessation: a randomized, double-blind, placebo-controlled trial. J Psychopharmacol. 2017;31(10):1377-79.

27 Andersen JV, Markussen KH, Jakobsen E, Schousboe A, Waagepetersen HS, Rosenberg PA, et al. Glutamate metabolism and recycling at the excitatory synapse in health and neurodegeneration. Neuropharmacology. 2021;196:108719.

28 Aryal SP, Fu X, Sandin JN, Neupane KR, Lakes JE, Grady ME, et al. Nicotine induces morphological and functional changes in astrocytes via nicotinic receptor activity. Glia. 2021;69(8):2037-53.

29 Lim DK, Kim HS. Opposite modulation of glutamate uptake by nicotine in cultured astrocytes with/without cAMP treatment. Eur J Pharmacol. 2003;476(3):179-84.

30 Namba MD, Kupchik YM, Spencer SM, Garcia‐Keller C, Goenaga JG, Powell GL, et al. Accumbens neuroimmune signaling and dysregulation of astrocytic glutamate transport underlie conditioned nicotine‐seeking behavior. Addict Biol. 2020;25(5):e12797.

31 Ciccarelli O, Wheeler-Kingshott C, McLean M, Cercignani M, Wimpey K, Miller D, et al. Spinal cord spectroscopy and diffusion-based tractography to assess acute disability in multiple sclerosis. Brain. 2007;130(8):2220-31.

32 Inglese M, Li BS, Rusinek H, Babb JS, Grossman RI, Gonen O. Diffusely elevated cerebral choline and creatine in relapsing‐remitting multiple sclerosis. Magn Reson Med. 2003;50(1):190-95.

33 Vrenken H, Barkhof F, Uitdehaag B, Castelijns J, Polman C, Pouwels P. MR spectroscopic evidence for glial increase but not for neuro‐axonal damage in MS normal‐appearing white matter. Magn Reson Med. 2005;53(2):256-66.

34 Chang L, Ernst T, Hillaire CS, Conant K. Antiretroviral treatment alters relationship between MCP-1 and neurometabolites in HIV patients. Antivir Ther. 2004;9(3):431-40.

35 Lentz MR, Westmoreland SV, Lee V, Ratai EM, Halpern EF, González RG. Metabolic markers of neuronal injury correlate with SIV CNS disease severity and inoculum in the macaque model of neuroAIDS. Magn Reson Med. 2008;59(3):475-84.

36 Meyerhoff D, Bloomer C, Schuff N, Ezekiel F, Norman D, Clark W, et al. Cortical metabolite alterations in abstinent cocaine and cocaine/alcohol‐dependent subjects: proton magnetic resonance spectroscopic imaging. Addict Biol. 1999;4(4):405-19.

37 Valcour V, Chalermchai T, Sailasuta N, Marovich M, Lerdlum S, Suttichom D, et al. Central nervous system viral invasion and inflammation during acute HIV infection. J Infect Dis. 2012;206(2):275-82.

38 Bokemeyer M, Ding X, Goldbecker A, Raab P, Heeren M, Arvanitis D, et al. Evidence for neuroinflammation and neuroprotection in HCV infection-associated encephalopathy. Gut. 2011;60(3):370-77.

39 Forton DM, Thomas HC, Murphy CA, Allsop JM, Foster GR, Main J, et al. Hepatitis C and cognitive impairment in a cohort of patients with mild liver disease. Hepatology. 2002;35(2):433-39.

40 Pfyffer D, Wyss PO, Huber E, Curt A, Henning A, Freund P. Metabolites of neuroinflammation relate to neuropathic pain after spinal cord injury. Neurology. 2020;95(7):e805-e14.

41 Mueller C, Lin JC, Sheriff S, Maudsley AA, Younger JW. Evidence of widespread metabolite abnormalities in Myalgic encephalomyelitis/chronic fatigue syndrome: assessment with whole-brain magnetic resonance spectroscopy. Brain Imaging Behav. 2020;14(2):562-72.

42 Brody AL, Gehlbach D, Garcia LY, Enoki R, Hoh C, Vera D, et al. Effect of overnight smoking abstinence on a marker for microglial activation: a [11C] DAA1106 positron emission tomography study. Psychopharmacology. 2018;235(12):3525-34.

43 Brody AL, Hubert R, Enoki R, Garcia LY, Mamoun MS, Okita K, et al. Effect of cigarette smoking on a marker for neuroinflammation: a [11C] DAA1106 positron emission tomography study. Neuropsychopharmacology. 2017;42(8):1630-39.

44 Bagory M, Durand-Dubief F, Ibarrola D, Comte J-C, Cotton F, Confavreux C, et al. Implementation of an Absolute Brain 1H-MRS Quantification Method to Assess Different Tissue Alterations in Multiple Sclerosis. IEEE Trans Biomed Eng. 2011;59(10):2687-94.

45 Fernando M, O'brien J, Perry R, English P, Forster G, McMeekin W, et al. Comparison of the pathology of cerebral white matter with post‐mortem magnetic resonance imaging (MRI) in the elderly brain. Neuropathol Appl Neurobiol. 2004;30(4):385-95.

46 Kirov II, Patil V, Babb JS, Rusinek H, Herbert J, Gonen O. MR spectroscopy indicates diffuse multiple sclerosis activity during remission. J Neurol, Neurosurg Psychiatry. 2009;80(12):1330-36.

47 Harezlak J, Buchthal S, Taylor M, Schifitto G, Zhong J, Daar E, et al. Persistence of hiv− associated cognitive impairment, inflammation and neuronal injury in era of highly active antiretroviral treatment. AIDS. 2011;25(5):625.

48 Letendre SL, Zheng JC, Kaul M, Yiannoutsos CT, Ellis RJ, Taylor MJ, et al. Chemokines in cerebrospinal fluid correlate with cerebral metabolite patterns in HIV-infected individuals. J Neurovirol. 2011;17(1):63-69.

49 Quarantelli M. MRI/MRS in neuroinflammation: methodology and applications. Clin Transl Imaging. 2015;3(6):475-89.

Mescher M, Merkle H, Kirsch J, et al. Simultaneous in vivo spectral editing and water suppression. NMR Biomed 1998;11:266-72.

Maddock R. ^1^H-MRS measurement of brain glutamate and its neurobiological meaning. UCLA Brain Research Institute MRS Affinity Group Seminar Series. April 13, 2022.


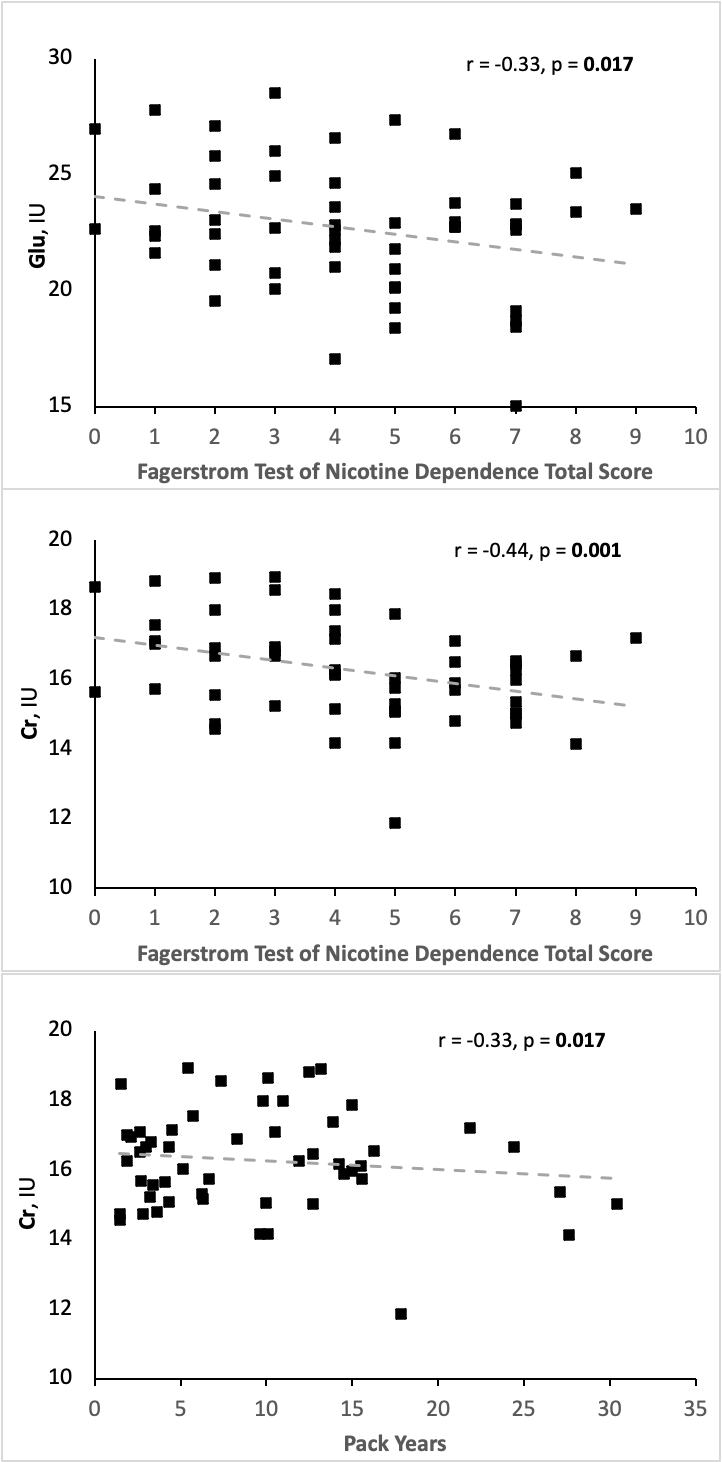
**Fig. S1 Relationships of clinically relevant smoking variables to MRS neurometabolite levels in dorsal anterior cingulate cortex (dACC) in the Smoking group.** Dashed lines indicate linear least-squares fits. (**upper**) glutamate (Glu) was lower for higher tobacco dependence (FTND Total Score). (**middle**) creatine+phosphocreatine (Cr) was also lower for higher FTND (*r* = -0.44, *p* = 0.001). (**lower**) Cr was lower for greater long-term tobacco exposure (pack years) (*r* = -0.33, *p* = 0.017). All tests Pearson partial correlation controlling for sex and age. Data for each participant (filled squares) are mean values in the dACC from the Time 1 and Time 2 scans. *IU* Institutional Units. *FTND* Fagerström Test for Nicotine Dependence.

| **Table S1.** Characteristics and clinical measures of Heavier Smoking subsample | |
| --- | --- |
| *N* | 29 |
| Age (years) | 34.2 ± 7.0 |
| male/female | 16/13 |
| Education (years) | **14.0 ± 2.1** |
| Mother’s education (years) | 13.9 ± 2.4 |
|  |  |
| FTND Total Score | 5.1 ± 1.9 |
| cigarettes/day | 15.0 ± 4.3 |
| pack-years | 12.3 ± 7.8 |
|  |  |
| Alcohol use (days/month) | **7.3 ± 8.1** |
| Cannabis use (days/month) | 6.0 ± 9.5 |

All participants in the Smoking sample reported smoking

≥ 5 cigarettes/day; of those, participants in the “Heavier

Smoking” subsample reported smoking ≥ 10 cigarettes/day

and had respiratory CO ≥10 ppm at intake.

Statistically significant *p* <0.05 values are in **bold**,

independent T-test vs. Nonsmoking sample.

*FTND* Fagerström Test for Nicotine Dependence.

| **Table S2.** LMM analysis of dACC neurometabolite levels (mean ± sd) for Heavier Smoking subsample. | | | | |
| --- | --- | --- | --- | --- |
|  | **Time 1** | **Time 2** |  |  |
| *N* | 26 | 21 |  |  |
| gray matter (volume %) | 52.2 ± 5.4 | 52.2 ± 4.7 |  |  |
| white matter (volume %) | 34.1 ± 6.2 | 33.4 ± 6.2 |  |  |
| CSF (volume %) | 13.7 ± 5.5 | 14.4 ± 4.9 |  |  |
|  |  |  |  |  |
| NAA (IU) | **24.0 ± 2.6^a^** | **23.7 ± 2.9** |  |  |
| Glu (IU) | **23.0 ± 4.2 ^a^** | **22.7 ± 2.6** |  |  |
| Cr (IU) | **16.4 ± 1.6 ^b^** | **16.2 ± 1.9** |  |  |
| Cho (IU) | **4.0 ± 0.6 ^a^** | **4.0 ± 0.5** |  |  |
| mI (IU) | 12.2 ± 2.5 | 13.1 ± 2.3 |  |  |

For all metabolite levels there were no significant within-sample differences between Time 2 and Time 1 on linear mixed model (LMM) accounting for sex and age.

^a^*p* <0.001, ^b^*p* <0.01 Heavier Smoking vs Nonsmoking on LMM accounting for sex and age.

Statistically significant *p* <0.01 values are in **bold**.

*dACC* dorsal anterior cingulate cortex. Time 1: after overnight abstinence, Time 2: 25-55 min after the first cigarette of the morning; *NAA* *N*-acetyl compounds, *Glu* glutamate, *Cr* creatine+phosphocreatine, *Cho* choline-compounds, *mi* *myo*-inositol, *IU* Institutional Units.

| **Table S3.** Associations of dACC MRS neurometabolites with tobacco dependence in Heavier Smoking subsample. | | | | | | | | | |
| --- | --- | --- | --- | --- | --- | --- | --- | --- | --- |
|  | **Fagerström Test**  **of Nicotine Dependence** | | | **Cigarettes per Day** | | | Pack Years | | |
|  | **df** | **R** | **p** | **df** | **R** | **P** | **df** | **r** | **p** |
| NAA | 25 | -0.34 | 0.089 | 25 | -0.33 | 0.091 | 25 | -0.29 | 0.142 |
| Glu | **24** | **-0.54** | **0.004** | 24 | -0.33 | 0.100 | 24 | -0.28 | 0.166 |
| Cr | **24** | **-0.58** | **0.002** | **24** | **-0.44** | **0.024** | **24** | **-0.49** | **0.012** |
| Cho | 25 | -0.32 | 0.100 | 25 | -0.27 | 0.169 | 25 | -0.27 | 0.169 |
| mI | 25 | -0.30 | 0.125 | 25 | -0.22 | 0.277 | 25 | -0.09 | 0.667 |

Results are for Pearson correlation partialling sex and age within the Heavier Smoking subsample.

Statistically significant *p* <0.05 values are in **bold**.

*dACC* dorsal anterior cingulate cortex, *MRS* magnetic resonance spectroscopy, *FTND* *NAA* *N*-acetyl compounds, *Glu* glutamate, *Cr* creatine+ phosphocreatine, *Cho* choline-compounds, *mI* *myo*-inositol.
