# Supplementary figures and images for: Smoking, tobacco dependence, and neurometabolites in the dorsal anterior cingulate cortex
Source: Mol Psychiatry. 2023 Sep 25;28(11):4756–65. doi: 10.1038/s41380-023-02247-0 (PMC10914613; doi:10.1038/s41380-023-02247-0)

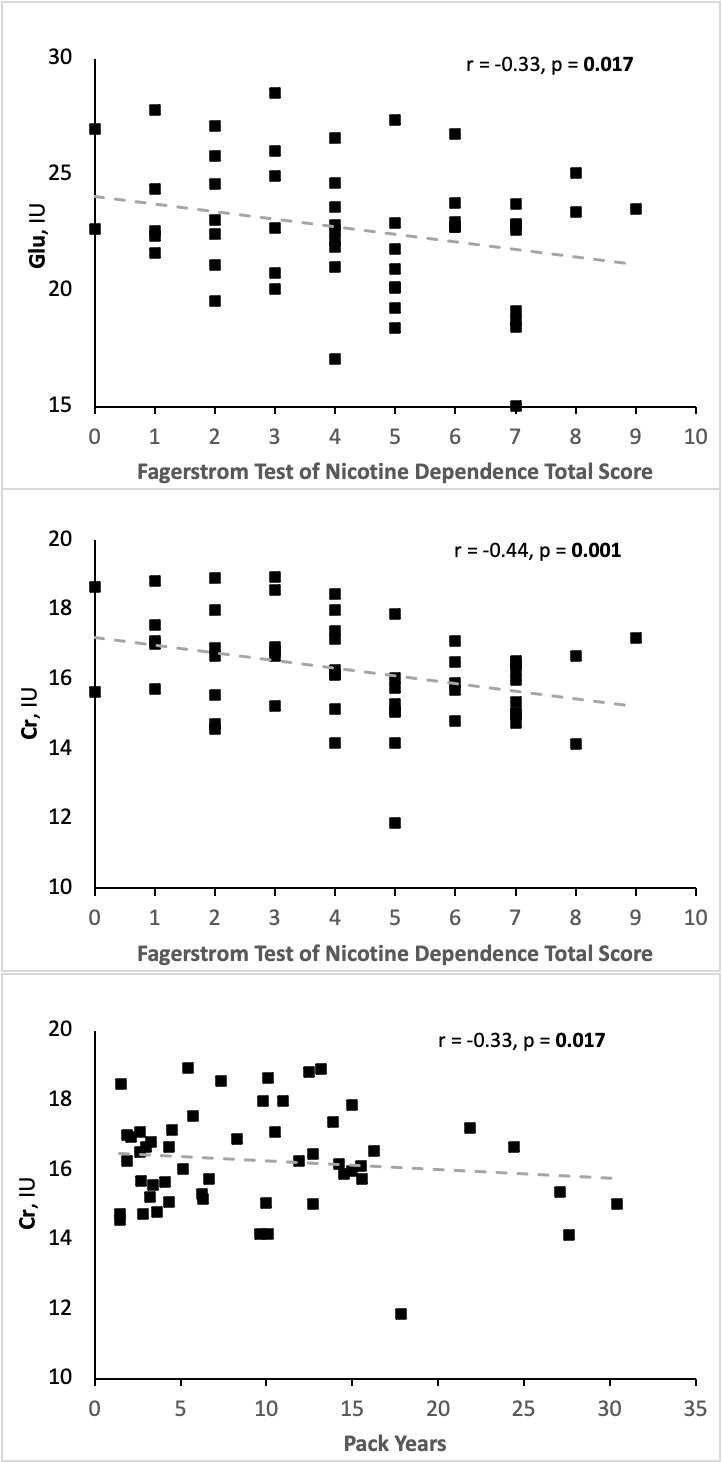

Supplement: Supplementary file 2 — Spplemental Figure S1 [file 41380_2023_2247_MOESM2_ESM.png]
